# A Scoping Review of Assessment Methods Following Undergraduate Clinical Placements in Anesthesia and Intensive Care Medicine

**DOI:** 10.3389/fmed.2022.871515

**Published:** 2022-04-05

**Authors:** Enda O'Connor, Evin Doyle

**Affiliations:** ^1^Department of Anesthesia and Intensive Care Medicine, St James's Hospital, Dublin, Ireland; ^2^School of Medicine, Trinity College, Dublin, Ireland

**Keywords:** intensive care, undergraduate education best practices, assessment and education, anesthesia, scoping review methodology

## Abstract

**Introduction:**

Anesthesia and intensive care medicine are relatively new undergraduate medical placements. Both present unique learning opportunities and educational challenges to trainers and medical students. In the context of ongoing advances in medical education assessment and the importance of robust assessment methods, our scoping review sought to describe current research around medical student assessment after anesthesia and intensive care placements.

**Methods:**

Following Levac's 6 step scoping review guide, we searched PubMed, EMBASE, EBSCO, SCOPUS, and Web of Science from 1980 to August 2021, including English-language original articles describing assessment after undergraduate medical placements in anesthesia and intensive care medicine. Results were reported in accordance with PRISMA scoping review guidelines.

**Results:**

Nineteen articles published between 1983 and 2021 were selected for detailed review, with a mean of 119 participants and a median placement duration of 4 weeks. The most common assessment tools used were multiple-choice questions (7 studies), written assessment (6 studies) and simulation (6 studies). Seven studies used more than one assessment tool. All pre-/post-test studies showed an improvement in learning outcomes following clinical placements. No studies used workplace-based assessments or entrustable professional activities. One study included an account of theoretical considerations in study design.

**Discussion:**

A diverse range of evidence-based assessment tools have been used in undergraduate medical assessment after anesthesia and intensive care placements. There is little evidence that recent developments in workplace assessment, entrustable activities and programmatic assessment have translated to undergraduate anesthesia or intensive care practice. This represents an area for further research as well as for curricular and assessment developments.

## Introduction

The inclusion of anesthesia and intensive care medicine (ICM) in undergraduate medical student placements is a relatively new development (1). Recent publications have sought to define suitable curricula in these disciplines (2, 3). With expanding placement opportunities come an ever-increasing obligation to ensure that student learning is effective and efficient, that student time is “well-spent,” and that we “maximize assessment for learning while at the same time arriving at robust decisions about learner's progress” (4).

The ICU and the anesthetic room can be challenging areas for student learning. Opportunities for history-taking and clinical examination are variable (1, 5). Patients undergoing anesthesia require a focused history and examination tailored to the upcoming anesthetic and surgical procedure (5). ICM patients are commonly sedated and/or confused, impeding history-taking. Clinical examination in the intensive care unit (ICU) is more challenging in the context of an immobile, unresponsive patient on extracorporeal devices (dialysis, mechanical ventilation). Furthermore, in both disciplines, procedural learning is often limited by the complex, high-stakes, time-sensitive aspects of common tasks (1, 5).

Conversely, anesthesia and ICM share learning opportunities not readily available during other placements. They are ideal environments for the vertical integration of primary and clinical sciences (6). Many learning topics are unique to these disciplines (e.g., acute respiratory distress syndrome, clinical brainstem death evaluation, inhalation anesthesia, pharmacological neuromuscular blockade). Other key elements of their curricula (e.g., the management of acute respiratory failure, shock, acute airway emergencies, sedation administration) are generic, high-stakes, transferrable clinical skills that could be viewed as important competencies for all doctors.

Current evidence suggests that ICM and anesthesia placements can achieve effective student learning outcomes (7). Nonetheless, the unique nature of their curricula may require a bespoke approach to learner assessment. Furthermore, valid and reliable tools are central to assessment decisions regarding high stakes competencies such as effective acute and perioperative patient care. Despite this, three recent papers on curriculum and effective teaching in the ICU and anesthetic room make no recommendations about student assessment (2, 3, 5). The first objective of our review therefore was to evaluate the nature and robustness of published assessment strategies in these high-stakes clinical specialties, incorporating an analysis of the theoretical bases for these publications.

The expansion of undergraduate anesthesia and ICM placements has occurred contemporaneously with an evolution in medical education assessment. Accordingly, practice is moving away from evaluating low-level cognitive learning objectives such as knowledge and understanding (using MCQs, written examinations) toward knowledge application (using extended matching questions, OSCEs) and most recently to clinical performance, either in a simulated or workplace environment (8–11). Furthermore, longitudinal methods such as programmatic assessment have in recent years gained in popularity (12). The extent to which this evolution has translated to assessment in undergraduate anesthesia and ICM education was the second objective of our review.

A scoping review methodology was used for two reasons. First, the authors had prior knowledge of the research topic and recognized that the range of published literature was unlikely to yield research of sufficient quality to enable a systematic review or a meta-analysis. Second, in light of recent advances in assessment practice, we anticipated a knowledge and/or research gap in the areas of anesthesia and ICM assessment. Our study methodology therefore needed to be tailored to identifying these gaps were they to exist (13, 14).

## Methods

We used the 6-step adaptation of Arksey and O'Malley's (15) scoping review framework as proposed by Levac et al. (13). These steps are (1) identifying research questions, (2) identifying relevant articles, (3) study selection, (4) charting the data, (5) collating, summarizing, and reporting the results and (6) consulting with stakeholders. In addition, we applied a scoping review quality checklist to enhance the rigor of our findings (16). The overarching purposes of our scoping review were (a) to describe the nature of existing research about undergraduate assessment after anesthesia/ICM placements and (b) to identify research gaps in this area.

The following six steps were applied.

### 1. Identifying Research Questions

The scoping research questions were:

1. What methods and practices of assessment have been reported in the literature for students undertaking clinical placements in anesthesia and/or intensive care medicine?
2. What educational theories have been articulated for the assessment methods published in the literature?

### 2. Identifying Relevant Articles

**Figure 1:**
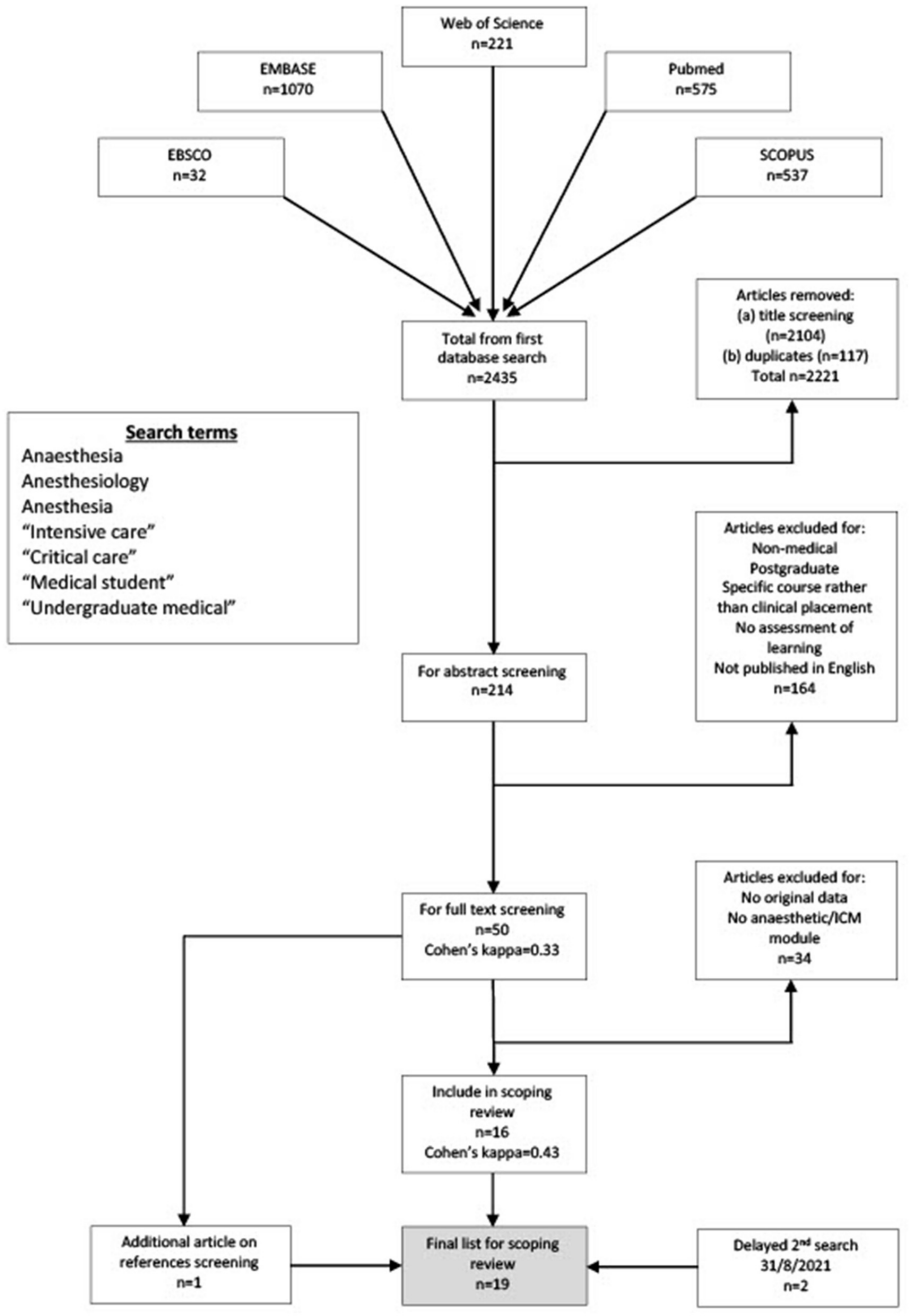
PRISMA flow diagram for the scoping review process. (ICM, intensive care medicine).

Using five online databases (EMBASE, SCOPUS, EBSCO, PubMed, Web of Science) we conducted a search for all available papers from January 1980 to 31/12/2020, using the search terms “medical student,” and several variations on “anesthesia” and “intensive care” to account for differences in regional terminology (see Figure 1). A librarian was used to assist with accessing articles. Reference lists of relevant articles were also included in the search. Due to the pandemic, the high intensive care and anesthesia workload in early 2021 led to a reallocation of research to clinical time, delaying the completion of the scoping review. Accordingly, a further search was performed up to 31/08/2021.

To be included, studies had to describe original research using an assessment tool following an undergraduate placement in anesthesia and/or intensive care medicine. Studies were excluded if they were not published in English, if they enrolled postgraduate or non-medical learners, or if the assessment followed a standalone courses rather than a clinical placement.

### 3. Study Selection

The three stages of study selection were based on title, abstract, and full-text searches respectively. Mendeley© software was used. The two authors independently screened the publications for study inclusion. A Cohen's kappa coefficient was calculated to quantify author agreement at each stage of study selection. The authors met regularly to discuss and resolve any disagreements about study inclusion. The number of studies included in each stage of selection, and the exclusion criteria are shown in the flowchart in Figure 1.

### 4. Charting the Data

Evaluating each study involved a combination of numerical description and general thematic analysis. For the former, the following information was extracted from each article: lead author; country of authorship; journal title; the year of publication; study design; number of research sites; sample size; assessment tools used; quantitative outcomes; Miller's learning outcomes (17); MERQSI score (18). Through thematic analysis, other details about the studies were recorded, including qualitative outcomes, important author's quotes, theoretical considerations and any insights pertinent to the research area. In accordance with Levac et al. (13) scoping review methodology, the 2 authors met following data extraction of the first 6 articles to determine whether the approach was “consistent with the research question and purpose” (page 4). Study authors were contacted directly if further information or clarification about their findings were deemed appropriate.

### 5. Collating, Summarizing and Reporting the Results

The information drawn from each article was summarized and tabulated (see Table 1).

**Table 1:** Summary of studies included in the scoping review.

**Reference** **Country**	**Study title**	**Journal** **Year** **Specialty site: single or multi-medical schools** **Assessment or Education**	**Study design** **Single arm or not** **Main data type(s)**	**Sample size** **Description of intervention** **Data collection tool(s)**	**Key messages from study findings Miscellaneous**	**Levels of learning outcomes assessed** **Educational theory if described** ***Validity/Reliability?***
Critchley et al. (19) Hong Kong	An adaptation of the objective structured clinical examination to a final year medical student course in anesthesia and intensive care	Anesthesia 1995 Anesthesia/ICM Single school Assessment	Descriptive account of 4 academic years – observational Single group having OSCE (no control) Quantitative (student performance and course feedback)	466 students in 4 years. Learning curriculum in place 4 week rotation → OSCE adaptation for skills, knowledge and communication. Summative (for final exam). Criterion-based marking Student survey/OSCE exam results	No student failed exam based on OSCE. Different domains of learning assessed in one examination Students perceived it a fair format (equally with other formats) but more stressful Assessment mapped to learning objectives derived from curriculum (knowledge and skills)	Miller's “Shows how” Assesses performance in simulated environment.
Lofaro et al. (20) US	An innovative course in surgical critical care for second-year medical students	Academic Medicine 1994 ICM Single school Education	Description of 12-week SICU rotation in 1990/91 Single arm, no control group Quantitative (rudimentary)	13 “sophomore” students 12 week SICU rotation Ax: “A shelf test from SCCM” (postgrad equivalent - ?format) Course evaluation by students	No student got <70% in SCCM test Student ratings were “uniformly high” Good description of range of teaching and learning methods	Miller's “Knows how” Contextual learning theory *Reliabilty/Validity NO but used an Society of Critical Care Medicine “Shelf” test*
Moll-Khosrawi et al. (21) Germany	Anaesthesiology students' Non-Technical skills: development and evaluation of a behavioral marker system for students (AS-NTS)	BMC Medical Education 2019 Anesthesia/EM Single school Education	4-steps of literature RV, focus groups/interviews, field observation and implementation/ validation Quant and qual	98 simulation activities with groups of 3 students (?yr, ?total number) Study describes the design of AS-NTS tool and evaluated feasibility, validity and inter-rater reliability of it Not summative Ax	Assessing 3 NTS: planning tasks, teamwork, and team orientation New Ax tool was feasible, valid and had good inter-rater reliability No Ax of technical skills or knowledge	Miller's “Shows how” for non-technical skills *Reliability YES* *Validity YES* *Intraclass correlation and Cohen's kappa* *Content validity index* *High validity and reliability*
Shams et al. (22) Saudi Arabia	Assessment of current undergraduate anesthesia course in a Saudi University	Saudi J Anesthesia 2013 Anesthesia/ICM Single school Assessment	Comparison of 3 different Ax tools (MCQs, portfolios and OSCEs) 3 Ax tools (quant) Short essays as well Student evaluation of course (quant)	154 students on 5-week OR/Anesthesia rotation described 20 question MCQ (validity and reliability) OSCE 6 stations Portfolio of presentations, logbook and trainer feedback (marks for each aspect) Correlation with final mark	Strong correlation between 3 Ax tools, and between each Ax tool and the overall outcome of the examination (r coeff >0.85 for all 3 tools). The MCQ exam was the highest predictor of overall exam mark, followed by OSCE and then portfolio.	Miller's “Shows how” *Reliability/Validity YES Cronbach alpha* *Cohen's kappa coefficient*
Morgan and Hogg (23) Canada	Evaluation of medical students' performance using the anesthesia simulator	Medical Education 2000 Anesthesia Single school Assessment	Pilot study, single arm interventional study, no control Faculty training to try to standardize Quantitative inter-rater reliability intra-class coefficient correlation between different Ax tools (Pearson) student evaluation	2 week anesthesia rotation with apprenticeship model in OR 24 students had sim assessment (to test reliability) and to compare with clinical and written exams for the rotation 6 scripted case scenarios linked to the learning objectives and outcomes of the anesthetic course	Strong IRR (ICC 0.87) Poor correlation between sim & written exam and between sim & clinical evaluation Better correlation between clinical & written exams Reliable assessment even if raters have not seen students during the rotation High student evaluation scores Authors did not expect written and sim to be well correlated but did hope for this between clinical and sim exams.	Miller's “Shows how” in simulated setting correlated with knows and knows how *Reliability YES* *Intraclass coefficient* *High inter-rater reliability*
Hamid et al. (24) Canada	The lack of construct validity when assessing clinical clerks during their anesthesia rotations	Can J Anesthesia 2020 Anesthesia Single school Assessment	Observational single arm study Quantitative (2 different numerical exams – anesth 1-10 and MCCQE part 1)	205 medical students undergoing 2 week anesthesia rotation Daily clinical evaluation (1-10 scale for 8 domains of medical competence) averaged over the 2 weeks period (average of 9 total daily evaluations per student)	No score of 1-4 (5=meets expectations) Mean score 7.1 DFA (discrimant function analysis) showed that anaes scores could identify weakest clerks “Failure to fail” phenomenon shown. A clear results of having subjective, workplace environment Ax tool Call for more valid, reliable and predictive assessments	Miller's “Does” observed in the workplace *Validity YES* *Discriminant Factor Analysis*
Morgan et al. (25) Canada	High-fidelity patient simulation: validation of performance checklists	BJA 2004 Anesthesia Single school Assessment	Quantitative Student scores on Sim Student feedback Single arm study	135 students 2 week anaesth rotation 1 day of Sim during this. 2 independent raters (0.97) 10 management Sim scenarios based on critical event [See (31)]. Wide faculty RV to decide “expected” and “critical performance items” (80% cutoff for inclusion) Internal consistency (compared with end of year clinical and anaes examination mark)	85% students agreed/strongly agreed the scenarios reflected rotation's learning objectives. Good face and content validity 5 of 10 scenarios had good internal consistency	Miller's “Shows how” *Reliability/Validity YES* *Cronbach's alpha* *Item analysis* *Variable validity (50% of scenarios had satisfactory validity and internal consistency)*
Rogers et al. (26) US	Quantifying learning in medical students during a critical care medicine elective: a comparison of 3 evaluation instruments.	CCM 2001 Intensive Care Medicine Single school Assessment	One student cohort with pre-/post- data collection. Ax tools randomized Quantitative data only Marks scored on 3 Ax tools	24 medical student volunteers 1 months ICM placement Ax using written exam (MCQs), OSCE and simulation (pre- and post-elective). All students received all 3 assessments at both time-points (order randomized)	Written test scores did not correlate with students' ability to perform (Sim) or apply their knowledge (OSCE). Scores reduced moving from knows, to knows how to does. Knowledge does not imply skills. Written tests overestimate achieving learning objectives OSCE has “grouping” or “compartmentalisation” of learning. Also, they are presented info rather than having to collect/deduce it themselves. Sim applies it all at once, thereby is harder.	Miller's “Shows how” *Validity discussion but no calculations*
Skinner et al. (27) UK	The use of computerized learning in intensive care: an evaluation of a new teaching program	Medical Education 1983 Medical ICM Single school Education	RCT of computer-assisted learning in addition to standard learning Quantitative Qualitative questionnaire	28 students (14 in each of 2 groups) Old-fashioned computer programme MCQ test pre- and post- learning.	From similar baseline, CAL showed 2-3 times higher post-test scores Computer familiarity associated with higher post-test scores Early study of eLearning/TeL Study focuses more on the learning rather than the assessment	A qualified doctor is “a highly complex blend of knowledge, attitudes and skills”(p53) Miller's “Knows”
Sharma and White (28) Canada	The use of multi-source feedback in assessing undergraduate students in a general surgery anaesthesiology clerkship	Medical Education 2010 (abstr) Anaesthesiology Single school Assessment	Feasibility study using MSF for student Ax Single arm Basic quant and qual data	Uncertain size (“groups of 20-24 students”). MSF using physicians, nurses, peers, patients and administrators 1-page MSF summary for each student	Each student had average of 25 assessments over 6 weeks. Concerns raised about validity of MSF assessments	Miller's “Does” Authors contacted but no response *Validity YES but no calculations*
Critchley et al. (29) HK	Web-based formative assessment case studies: role in a final year medicine 2-week anesthesia course	Anesthesia and Intensive Care 2009 Anesthesia Single school Assessment	Quantitative Feedback qualitative Single arm study – no control or comparator group No pre-/post- data collection	2 week anesthesia rotation Ax: 40-item MCQ and 2 written case reports 149 volunteer students with 6 online FACS (81% used it) Use of FACS during 2006-2007, with milestone MCQs integrated in each case Login and participation details MCQ scores in the FACS	Wide variation in FACS usage, time spent on each FACS. FACS comparable to in-class teaching on feedback Weak correlation between FACS usage and summative MCQs, but stronger correlation with written case reports.	Miller's “Knows how” Low stakes formative
Morgan et al. (30) Canada	Validity and Reliability of undergraduate performance assessments in an anesthesia simulator	CJA 2001 Anesthesia Single school Assessment	Single arm study of students doing simulation Quantitative data (student performance scores, validity and reliability calculations)	140 students 10 day anesthesia rotation with simulator on Day 8 Faculty “marked” students on sim performance, each marking 25-34 students 25-point criterion-based marking form Reliability and validity assessed	High inter-rater reliability in the faculty assessments Poor correlation with students' final clinical and written exam results Concerns about low validity of assessment Low internal consistency of checklist assessments	Knowledge vs “hands-on medical management problems” (p230) Miller's “Shows how” *Validity/reliability YES* *Intraclass coefficient* *Item-total correlation coefficient* *Acceptable reliability with 2 assessors*
Rogers et al. (7) US	Medical Students can learn the basic application, analytic, evaluative and psychomotor skills of critical care medicine	CCM 2000 ICM Single school Education/Assessment	Pre- and Post- elective design using 2 clinical scenarios at 5 OSCE stations (randomized to be pre- or post-) Small randomized control group (n=3) All quantitative	1 month CCM elective OSCE Ax tool on D1 and last day of elective 2 independent assessors of videotaped OSCEs Pre-/Post- comparison	40 students doing elective 3 students not doing elective Only 27 videos were usable 80% of assessments had adequate reliability Consistent improvement in pre- and post-elective scores (except OSCE I = initial patient assessment)	Written exams reliable but not valid (if “we are training students to perform”) *Reliability/Validity YES Kappa coefficient* *Good reliability for 80% of tools used* Miller's “Shows How”
Morgan et al. (31) Canada	Identification of gaps in the achievement of undergraduate anesthesia educational objectives using high fidelity patient simulation	Anaesth Analges 2003 Anesthesia Single school Education/Assessment	Single arm No control One data collection point Quantitative	135 students – 165 scenarios 2 week anesthesia course. 1 day simulation during this. Development of 10 Sim scenarios based with 3-8 learning items for each. Expert opinion about expected and critical items in these scenarios (80% minimum) Faculty score student performance in Sim sessions	“Expected performance criteria” of students vs “Critical management items” (p1690). 11 of 18 learning items performed by 75% of students. Sim effectively identified critical management items not performed Minimal focus on how teaching delivered. Sim = learning and assessment in one = assessment for learning.	*Reliability YES;* *Inter-rater reliability* *High reliability scores* Sim enables identification of “discrepancies between expected and actual educational outcomes” (p1694) Miller's “Shows how”
Leung et al. (32) HK	Evidence of virtual patients as a facilitative learning tool on an anesthesia course	AHSE 2015 Anesthesia Single school Education	Quantitative student scores Student feedback (Likert) Non-randomized 2x2 groups	VPs used to enhance learning. This is a study of learning, NOT assessment. Ax used: MCQs (60-item), SAQ paper, and “modified essay paper”	VPs improved assessment scores	Miller's “Knows How”
Kapur et al. (33) US	Implementation of a formal medical intensive care unit curriculum for medical students	AJRCCM 2019 (abstr) ICM Single school Education	Quantitative Student feedback	4 week MICU rotation Pre- and Post- rotation tests of knowledge and attitudes	Knowledge improved (67% → 81%) “Comfort” taking history and managing common ICU scenarios improved but no demonstration of this.	Miller's “Knows how” Contacted author but no response
Rogers et al. (34) US	Teaching medical students complex cognitive skills in the intensive care unit	CCM 1995 ICM Education/Assessment	Single group Pre-/Post- design Randomized crossover trial Quantitative	1 month SICU rotations 33 students ICU curriculum designed with PbL. Cognitive learning emphasized. Written examinations	Knowledge and application improved	Miller's “Knows How”
Ho et al. (19) US	Developing the eMedical Student (eMS) – a pilot project integrating medical students into the tele-ICU during the COVID-19 Pandemic and beyond	Healthcare 2021 ICM Education	Single group Non-randomized Pre-/post-test design	5 students 4 week rotation MCQ assessment	Improved knowledge on MCQ test	Miller's “Knows”
Gergen et al. (35) US	Integrated critical care curriculum for the third-year internal medicine clerkship	MedEdPortal 2021 ICM Education	Single group Non-randomized with pre-/post-test design	41 3^rd^ year students Seven sessions, over 4 weeks 15 Written short answer questions	Improved knowledge on SAQ test	*No specific reliability/validity but used standardized tests from American College of Physicians question bank* Miller's “Knows”

*ICM, intensive care medicine; OR, operating room; IRR, interrater reliability; MCCQE, Medical Council of Canada Qualifying Examination; Sim, simulation; OSCE, objective structured clinical examination; MCQ, multiple choice question; RCT, randomised controlled trial; MSF, multisource feedback; FACS, formative assessment case studies; VPs, virtual patients; MICU, medical intensive care unit; SICU, surgical intensive care unit; SAQ, short-answer question; EM, emergency medicine*.

### 6. Consulting With Stakeholders

Consultation was undertaken via email with 3 stakeholders involved in undergraduate education and/or anaesthesiology/intensive care medicine teaching, each working in a different academic institution. Preliminary study results were shared with them. The purpose of the consultation was to seek opinions about any omitted sources of study information, to gain additional perspectives on the study topics, and to invite opinions about the study findings.

## Results

A total of 2,435 results were returned from the initial search between the 5 databases, of which 17, published between 1983 and 2020, were selected for full-text review (6, 20–34, 36). The second search performed in August 2021 returned 2 further studies published in 2021 (19, 35). The findings of these 19 studies are shown in Table 1.

Of the 19 studies, 9 (47.4%) involved anesthesia (21, 23–25, 28–32), 8 (42.1%) intensive care medicine (6, 19, 20, 26, 27, 33–35) and 2 (10.5%) a combination of both disciplines (22, 36). The primary research focus was on the assessment instrument and on student learning in 9 (22–26, 28–30, 36) and 7 (19–21, 27, 32, 33, 35) studies respectively. The remaining 3 studies had equal research focus on learning and the assessment tool.

All were single-center studies, 14 of which (73.7%) were conducted in Canada, USA and Hong Kong. The average sample size across all studies was 119 students (range 5–466). Clinical placements lasted 2–12 weeks, with a median duration of 4 weeks. Fourteen studies (73.7%) had 2 or 4 week placements.

Twelve of 19 studies (63.2%) had a non-randomized design and collected assessment data at one timepoint only (20–25, 28–32, 36). Conversely, the remaining 7 studies (36.8%) were either RCTs and/or had a pre-/post-test study design (6, 19, 26, 27, 33–35). Despite all studies reporting solely or mainly quantitative data, none conducted a power analysis to evaluate the required sample size. Ten studies (52.6%) considered the issues of the reliability and/or validity of their assessment tools (6, 21–26, 28, 30, 31). Two additional studies used standardized assessment questions from the Society of Critical Care Medicine and the American College of Physicians (20, 35). The average MERQSI score was 12.3 (range 5–15) out of a maximum of 18.

### Q1: What Methods of Undergraduate Assessment Have Been Reported for Medical Students Undertaking Clinical Placements in Anesthesia and/or Intensive Care Medicine?

A wide variety of student assessment tools were used across the 19 included studies. These are shown in Table 2. The most common methods were multiple choice questions (7 studies; 36.8%) (19, 22, 26, 27, 29, 32, 35), written assessment (6 studies; 31.6%) (20, 22, 23, 29, 32, 34) and simulation (6 studies; 31.6%) (21, 23, 25, 26, 30, 31).

**Table 2:** Assessment tools used following clinical placements in Anaesthesia and Intensive Care Medicine.

**MCQs**: 7 studies (19, 22, 26, 27, 29, 32, 35)
**Written assessment (not including OSCEs, MCQs or correlation with end of year exams)**: 6 studies (20, 22, 23, 29, 32, 34)
**Simulation**: 6 studies (21, 23, 25, 26, 30, 31)
**OSCEs**: 4 studies (6, 22, 26, 36)
**Clinical assessment**: 3 studies (23, 25, 30)
**Other**: FACS 1 (29), Portfolio 1 (22), MSF 1 (28), SAQs 1 (32), Unknown (33)
**More than one assessment tool**: 7 studies (22, 23, 25, 26, 29, 30, 32)

*MCQs, multiple choice questions, OSCEs, objective structured clinical examinations; FACS, formative assessment case studies; MSF, multisource feedback; SAQs, short answer questions*.

Seven studies (36.8%) used a combination of more than one assessment tool (22, 23, 25, 26, 29, 30, 32), which in 3 studies included final end-of-year examinations (22, 25, 30).

All 7 studies with a pre-/post-test design showed an improvement in assessment outcomes after clinical placements. All had a 4 week/1 month clinical placement and all were in intensive care medicine. Only 2 of these 7 studies evaluated student performance using simulation and/or OSCE stations (6, 26). The remaining 5 studies evaluated student knowledge using MCQs or written assessment tools (19, 27, 33–35).

### Q2: What Educational Theories Are Evident in Studies of Undergraduate Medical Assessment After Anesthesia and/or Intensive Care Medical Placements?

Of the five studies with a primary research focus on student learning, contextual learning theory was used as the theoretical basis for one study (20). No other study made any methodological references to an underlying educational theory.

Though seldom articulated, Miller's Pyramid of learning outcomes was an important theoretical foundation in most of the studies (17). Nine studies (47.4% evaluated learning outcomes in the “shows how” level 3 domain using simulation (21, 23, 25, 26, 30, 31) and/or OSCEs (6, 22, 26, 36). Ten studies had learning outcomes in the “knows” or “knows how” domains, using a combination of MCQs, SAQs, essay questions or online case studies (19, 20, 22, 23, 26, 27, 29, 32, 34, 35). One study did not describe the assessment tool used (33). Finally, two studies evaluated student performance in the workplace using subjective observation (24) and multi-source feedback (28), thereby targeting Miller level 4 “does” learning outcomes. No study used workplace assessment tools (WPAs) to assess undergraduate learning outcomes.

## Discussion

Our scoping review illustrates the heterogeneity of literature around assessment in undergraduate anesthesia and intensive care medicine. Published studies used numerous assessment instruments targeting learning outcomes that were either knowledge-based (using selected-response MCQs and constructed-response written tests) or performance-based (using OSCEs or a simulated clinical environment). The findings of the review also attest to the meaningful undergraduate learning that can occur in these clinical settings.

Though heterogenous, a majority of the included studies used evidence-based assessment strategies insofar as either the chosen tools have strong evidence supporting their use in UGME (MCQs, written exams, simulation, OSCEs) or more than one method was used to inform assessment decisions (8, 9, 37). Furthermore, all except one study (33) used an assessment strategy appropriate to the learning outcomes mapped to Miller's pyramid.

A key objective of undergraduate medical education is to equip students with the competencies to deliver effective and safe patient care in their first year of medical practice and beyond. The assessment of knowledge, or the theoretical application of that knowledge alone may not be sufficient to judge whether students are equipped with those skills (38). Accordingly, 9 studies in our review adopted a competency-based approach and used OSCEs or simulation to evaluate student performance. Only 3 of these studies however used complementary tools to evaluate learning in the domains of knowledge and understanding as well as competence (22, 23, 26). These are the most informative studies in our review for educators making instructional design decisions about student assessment after anesthesia and ICM placements.

The most frequent tool used to evaluate student performance in our review was simulation, whereby learners were assessed in the “show how” learning domain. Performance in a simulated environment however correlated poorly with written assessments, suggesting a role for both tools in reaching a more complete assessment decision about a student's learning outcomes. There was scant evidence in the studies however of observed performance assessment *in the workplace*–the “does” domain of Miller's pyramid–which likely reflects a lack of active student work in ICUs and anesthetic rooms; students in these environments are more likely to learn by observing than by doing.

Nonetheless, recent trends in undergraduate assessment have led to greater emphasis on workplace performance and to the use of entrustable professional activities (EPAs) (39–41). EPAs entail assessors observing students performing “units of work” (42) (p2) thereby judging the level of supervision each student needs with that activity–the entrustment decision (11). They are mapped to learning curricula and are commonly informed by assessment in the workplace (11). To date, most EPA studies in UGME centre around internal medicine and general surgery. While we identified no studies using EPAs solely for the purposes of undergraduate anesthesia/ICM assessment, some include anesthesia or ICM placements as part of a broad learning curriculum (43–45). Furthermore, of the 13 undergraduate EPAs published by the Association of American Medical Colleges, 3 (e.g., recognize a patient requiring urgent or emergent care and initiate evaluation and management) have direct relevance to ICM and anesthesia (46). The use of EPAs also helps address long-standing concerns about graduating student's readiness to commence internship (47). A strong case can therefore be made for applying EPAs to anesthesia and ICM.

We did not identify any studies using a programmatic approach to assessment, though our literature search may not have found studies which included anesthesia or ICM as part of a broader programme-wide assessment strategy. Moreover, programmatic assessment challenges the “module-specific” nature of traditional UGME, viewing a clinical placement within a broader context of an overall curriculum (12). Therefore, it does not readily apply to our review, which *ab initio* focused on the assessment of a specific placement in anesthesia or ICM.

A common criticism of education research is that theoretical considerations are not brought to the fore. This also applies to the majority of articles in our review. Notwithstanding this, most of the included studies were designed in such a way that the use of a theoretical paradigm could be implied. Moreover, some of the studies used instructional design methodology. The primary use of technology to enhance learning in 3 studies (27, 29, 32) draws from eLearning theory (48). Adopting problem-based learning in 3 further studies acknowledged the importance of constructivism in effective education (6, 26, 34). Aspects of workplace learning theory were evident in the numerous studies that promoted learning within the operating theater and/or the intensive care unit (6, 20, 22, 23, 26, 29, 30, 33, 34, 36). The 6 studies using simulation for assessment (21, 23, 25, 26, 30, 31) likely reflected the importance of experiential learning theory and reflection (49).

A recent development that is difficult to ignore is the impact of the COVID-19 pandemic on undergraduate education. Accounts of formal assessment in the context of students working or learning during the pandemic appear to be rare (19). This likely reflects the time constraints and staff redeployment during the pandemic when clinical activities took precedence over faculty pursuits (50). Paradoxically, when viewed through the lens of Miller's pyramid, at a time when students may have been more actively involved in clinical care–in “doing” work–they were least likely to be formally assessed.

Our study therefore highlights a gap between published research in anesthesia/ICM assessment and recent advances in undergraduate assessment. However, for practice to change in these disciplines, the approach to student education in anesthetic rooms and ICUs must first evolve to allow more active student participation in daily clinical activities. Observed workplace assessment can only occur in environments where learners play a legitimate role in patient care. This is the main research gap identified in our review and is an important area for future research into the undergraduate study of anesthesia and intensive care medicine.

Our study results may be limited by the omission of some relevant articles. Each author however individually performed the search and results were then combined. Studies were individually evaluated for inclusion and though Cohen's coefficient showed a suboptimal correlation, author discussion at each step of study selection addressed any differences in opinion. We included any study where discussion did not resolve perceived differences. To improve the rigor of our study, we used the guidelines published by Maggio et al. in all stages of our review (16). We consulted with key external stakeholders but this step yielded no additional information.

In conclusion, our findings yield three useful insights. First, they act as a practice guide for educators directly involved in the design, delivery, and assessment of undergraduate learning in anesthesia and intensive care medicine. Second, they are informative for university educators tasked with the general organization and design of undergraduate medical education, helping them position anesthesia and intensive care medicine in strategies around programmatic assessment and workplace-based entrustable decision-making. Finally, they identify a large research gap for future studies to focus upon.

## Author Contributions

EO'C and ED were equally involved in all aspects of this study including design, literature searches, data collection, and manuscript writing. Both authors contributed to the article and approved the submitted version.

## Conflict of Interest

The authors declare that the research was conducted in the absence of any commercial or financial relationships that could be construed as a potential conflict of interest.

## Publisher's Note

All claims expressed in this article are solely those of the authors and do not necessarily represent those of their affiliated organizations, or those of the publisher, the editors and the reviewers. Any product that may be evaluated in this article, or claim that may be made by its manufacturer, is not guaranteed or endorsed by the publisher.
